# Development and validation of a nomogram for evaluating the prognosis of immunotherapy plus antiangiogenic therapy in non-small cell lung cancer

**DOI:** 10.1186/s12935-022-02675-y

**Published:** 2022-08-21

**Authors:** Hao Huang, Yao Chen, Xuezi Weng, Sirou Li, Lin Zhang, Peisong Chen

**Affiliations:** 1 Department of Laboratory Medicine, the First Affiliated Hospital of Sun Yat-Son University, Guangzhou, 510000 Guangdong China; 2 Department of Laboratory Medicine, Sun Yat-Sen University Cancer Center, Guangzhou, 510000 Guangdong China

**Keywords:** Immune checkpoint inhibitors, Antiangiogenic, Non-small cell lung cancer, Nomogram, Prognosis

## Abstract

**Background:**

With the combination therapy of PD-1/PD-L1 antibody and antiangiogenic drugs used widely in clinic, a novel method to estimate the prognosis of patients is needed. We aimed to develop a nomogram to examine prognosis of anti-PD-1/PD-L1 antibody plus bevacizumab in non-small cell lung cancer (NSCLC) patients.

**Methods:**

We developed a nomogram using the cohort involving 204 NSCLC patients who treated with immunotherapy and anti-angiogenesis therapy. The nomogram was validated under the same conditions in another cohort with 69 patients. Prognostic factors were analyzed by Cox regression analysis. The nomogram was internally validated using bootstrap resampling and then externally validated. Performance was assessed using concordance index, calibration curve and decision curve analysis. Clinical utility was evaluated using receiver operation characteristic curve.

**Results:**

Pleural metastasis (P = 0.001, HR = 2.980, 95%CI 1.521–5.837), ANC (P < 0.001, HR = 5.139, 95%CI 2.081–12.691), ALC (P = 0.010, HR = 0.331, 95%CI 0.142–0.771), B cells (P = 0.005, HR = 0.329, 95%CI 0.151–0.714), Treg cells (P = 0.002, HR = 2.934, 95%CI 1.478–5.826) were independent prognostic factors. The calibration curves showed good consistency and the C-index of nomogram were 0.808, 0.741 in training and external validation cohort, respectively. The area under the curve (AUC) in receiver operation characteristic curves (ROC) are 0.833 (P < 0.001) and 0.908 (P < 0.001), respectively.

**Conclusion:**

We build an accurate and convenient nomogram to predict long-time overall survival (OS) of NSCLC patients treated with PD-1/PD-L1 antibody and antiangiogenic drugs and validated this nomogram. The nomogram might be helpful to clinicians to estimate long-time OS of NSCLC patients treated with PD-1/PD-L1 antibody and antiangiogenic drugs.

## Background

Lung cancer is one of the most common diagnosed cancers and main causes of cancer -related death worldwide, representing approximately one in 10 (11.4%) cancers diagnosed and one in 5 (18.0%) deaths. The survival of patients with lung cancer at 5 years after diagnosis is only 10–20% in most countries during 2010 through 2014. Approximately, 80–85% patients of histological types diagnosed as non-small cell lung cancer (NSCLC) [1].

Compared with classical platinum-based chemotherapy or docetaxel, immune checkpoint inhibitors (ICIs), such as programmed cell death 1/programmed death ligand 1 (PD-1/PD-L1), for treatment of NSCLC demonstrated superior survival [2]. Up to now, Atezolizumab, Nivolumab and Pembrolizumab have been approved by FDA to treat NSCLC. Clinical trials have found that ICI therapy is effective for first-line and second-line treatments of advanced NSCLC, consolidated treatment of locally advanced NSCLC, and neoadjuvant treatment of early NSCLC [3]. Despite the promising efficacy of immunotherapy in NSCLC, the success of ICIs is currently limited to a small number of patients, with the overall response rate for each of these drugs is roughly 15–20% [1]. More efficient strategies are needed.

Since 1971, Judah Folkman proposed that blocking angiogenesis (e.g. antiangiogenesis) would be an effective anticancer therapy, bevacizumab, a monoclonal antibody directed against vascular endothelial growth factor (VEGF), has been approved of antiangiogenic therapy in NSCLC [4]. In trials of patients with recurrent or metastatic NSCLC, bevacizumab with standard pemetrexed/paclitaxel doublet therapy improved both progression-free survival (PFS) and overall survival (OS) compared chemotherapy only [5]. It shows that the combination of anti-angiogenesis therapy and ICI is very promising.

Recent years, adding bevacizumab to immunotherapy has become a common combined immunotherapy strategies in NSCLC treatment [6]. And some studies have shown that antiangiogenic therapy can improve the efficacy of PD-1/PD-L1 antibodies in cancer patients [7]. However, the benefit of the combination strategies in NSCLC treatment is not clear. We aimed to use common hematological indicators to develop a nomogram to quantify risk of progression and predict 5-year survival rate for patients.

## Materials and methods

### Patients

This retrospective study was conducted at Sun Yat-sen University Cancer Center between January 1, 2017 and December 31, 2021. Adult patients who were histologically confirmed NSCLC and treated with anti-PD-1/PD-L1 plus bevacizumab were enrolled. Patients received ICI therapy (nivolumab, pembrolizumab, or atezolizumab) previously were throw away. Besides, patients with incomplete information or lost follow-up were excluded. To examine the generalizability of the model, an external validation cohort was set at the First Affiliated Hospital of Sun Yat-son University. Totally 204 patients were included in primary cohort after selected by our inclusive and exclusive criteria. And 69 patients were enrolled in validate cohort. The authenticity of this article has been validated by uploading the key raw data onto the Research Data Deposit public plat form (www.researchdata.org.cn), with the approval RDD number as RDDA2020001591.

### Data collection

The clinical information was obtained from electronic medical record system (EMR) and experiment data was collected from laboratory information system (LIS). We collected demographics (age, gender, patient status, BMI, alcohol, smoking, TNM stage), histology, comorbidities (no. of metastatic sites, pleural metastasis, lung metastasis, brain metastasis, liver metastasis, bone metastasis), Blood Routine examination data (WBC, ANC, ALC, NLR, LMR, PLR, PLT). As for bevacizumab which inhibiting the production of new blood vessels may lead to bleeding in NSCLC, we collected some data of coagulation function (PT, APTT, Fbg, TT). Refer to ICI therapy, lymphocyte subsets especially T lymphocyte subpopulations (CD3 + , CD3CD4 + , CD3CD8 + , CD19 + , CD3−CD16 + CD56 + , CD4CD25, CD8CD25) were noticed.

Routine Blood Test was estimated by Sysmex XN 9000 (Japan), Coagulation Test was estimated by Sysmex XN 5100 (Japan), Lymphocyte Subsets Exam was estimated by BD FACS Canto II (USA).

### Statistics

ICI therapy in the study were measured as Atezolizumab, Nivolumab and Pembrolizumab. And antiangiogenic therapy refers in particularly to bevacizumab. ANC is absolute neutrophil count. ALC is absolute lymphocyte count. NLR is calculated by neutrophil–lymphocyte ratio. LMR is calculated by lymphocyte-monocyte ratio. PLR is calculated by platelet to lymphocyte ratio. The primary outcome index about prognosis was OS (overall survival), which was calculated as the duration between the date of diagnosis to death from any cause.

Continuous variables with normal distribution or non-normal both expressed as median and IQR. Categorical variables are showed in frequency and percentages Continuous variables with normal or abnormal distribution compared by the Students’ test or Mann–Whitney U-test. χ2 test or Fisher’s test were used to compare Categorical variables respectively. And risk factors were analyzed by cox logistic regression using variables with P < 0.05 and expressed as odds ratios (OR) and 95% confidence interval (CI). Kaplan–Meier method was used to estimate the survival.

A prediction model of prognostic was developed based on the risk factors identified in the cox regression analysis. Harrell’s C-index were evaluated to quantify the discrimination performance of the nomogram. The C-index value higher than 0.75 was thought to indicate a better degree of discrimination. The nomogram was constructed for predicting the prognostic of NSCLC patients treated with anti-PD-1/PD-L1 plus bevacizumab. The decision curve was plotted for the model of nomogram. Receiver operation characteristic (ROC) curve was used for analyzing the prediction value of the model with the area under curve (AUC).

During all the statistical analysis, variables with P < 0.05 were considered statistically significant. Data were analyzed with the IBM SPSS Statistics for Windows (version 24.0) and R software (version 3.1.4; http://www.Rproject.org).

## Results

### Patient characteristics

A total of 273 patients treated with anti PD-1/PD-L1 antibody plus bevacizumab from Sun Yat-sen University Cancer Center in 2018–2021 were included in this study: 204 in the training cohort and 69 in the validation cohort. Most patients in the training cohort were men (83.82%) and less than 59 years old (50.49%). Almost half of patients in the validation cohort were  < 59 years (34, 51.28%) and 71.00% (49/69) were men. Up to December 31, 2021, there were 47 (22.71%) patients died in training cohort and 15 (21.74%) in validating cohort. Most of the patients in both cohorts admitted smoking (> 50) and had healthy BMI (> 50), less patients were used to drink (< 20%). Quite a number in both cohorts had more than 2 metastases (> 30%), such as pleural metastasis, lung metastasis, brain metastasis, liver metastasis, bone metastasis and so on.

Only the sex and histology distribution differed significantly between the training and validation groups. The clinicopathological, characteristics and laboratory results of patients in the training and validation cohorts are listed in Table 1.

**Table 1:** Demographic, clinicopathological, and hematological index of NSCLC patients in the training and validation cohorts

Characteristic	Primary cohort No. (%)			Validation cohort No. (%)			P value
	All patients (204)	Survive (157)	Dead (47)	All patients (69)	Survive (54)	Dead (15)	
Age (median),* n* (%)							
< 59	103 (50.49%)	76 (37.25%)	27 (13.24%)	34 (49.28%)	26 (37.68%)	8 (11.59%)	0.86
≥ 59	101 (49.51%)	81 (37.71%)	20 (9.80%)	35 (50.72%)	28 (40.58)	7 (10.14%)	
Sex,* n* (%)							
Male	171 (83.82%)	134 (65.69%)	37 (18.14%)	49 (71.00%)	37 (53.62%)	12 (17.39%)	0.02
Female	33 (16.18%)	23 (11.27%)	10 (4.90%)	20 (29.00%)	17 (24.64%)	3 (4.35%)	
Patient status							
BMI,* n* (%)							
< 18.5	19 (9.31%)	7 (3.43%)	12 (5.88%)	3 (4.35%)	1 (1.45%)	2 (2.90%)	0.08
18.5–24	121 (59.31%)	91 (44.61%)	30 (14.71%)	35 (50.72%)	29 (42.03%)	6 (8.70%)	
> 24	64 (31.38%)	59 (28.92%)	5 (2.45%)	31 (44.93%)	24 (34.78%)	7 (10.14%)	
Alcohol,* n* (%)							
No	162 (79.41%)	123 (60.29%)	39 (19.12%)	56 (81.16%)	45 (65.22%)	11 (15.94%)	0.75
Yes	42 (20.59%)	34 (16.67%)	8 (3.92%)	13 (18.84%)	9 (13.04%)	4 (5.80%)	
Smoking,* n* (%)							
No	89 (43.63%)	70 (34.31%)	19 (9.31%)	38 (55.07%)	33 (47.83%)	5 (7.25%)	0.09
Yes	115 (56.37%)	87 (42.65%)	28 (13.73%)	31 (44.93%)	21 (30.43%)	10 (14.495)	
Histology,* n* (%)							
Adenocarcinoma	107 (52.45%)	83 (40.69%)	24 (11.76%)	48 (69.57%)	40 (57.97%)	8 (11.59%)	0.01
Squamous cell carcinoma	97 (47.55%)	74 (36.27%)	23 (11.27%)	21 (30.43%)	14 (20.29%)	7 (10.14%)	
No. of metastatic sites,* n* (%)							
< 2	141 (69.12%)	110 (53.92%)	21 (10.29%)	42 (60.87%)	35 (50.72%)	7 (10.14%)	0.21
≥ 2	63 (30.88%)	47 (23.04%)	26 (12.75%)	27 (39.13%)	19 (27.54%)	8 (11.59%)	
Pleural metastasis,* n* (%)							
Absent	149 (73.04%)	124 (60.78%)	25 (12.25%)	42 (60.87%)	38 (55.07%)	4 (5.80%)	0.05
Present	55 (26.96%)	33 (16.18%)	22 (10.78%)	27 (39.13%)	16 (23.19%)	11 (15.94%)	
Lung metastasis,* n* (%)							
Absent	140 (68.63%)	107 (52.45%)	33 (16.18%)	50 (72.46%)	39 (56.52%)	11 (15.94%)	0.55
Present	64 (31.37%)	50 (24.51%)	14 (6.86%)	19 (27.54%)	15 (21.74%)	4 (5.80%)	
Brain metastasis,* n* (%)							
Absent	176 (86.27%)	134 (65.69%)	42 (20.59%)	61 (88.41%)	46 (66.67%)	15 (21.74%)	0.65
Present	28 (13.73%)	23 (11.27%)	5 (2.45%)	8 (11.59%)	8 (11.59%)	0	
Liver metastasis,* n* (%)							
Absent	179 (87.75%)	140 (68.63%)	39 (19.12%)	61 (88.41%)	47 (68.12%)	14 (20.29%)	0.88
Present	25 (12.25%)	17 (8.33%)	8 (3.92%)	8 (11.59%)	7 (10.14%)	1 (1.45%)	
Bone metastasis,* n* (%)							
Absent	153 (95.70%)	117 (57.35%)	36 (17.65%)	50 (72.50%)	41 (59.42%)	9 (13.04%)	0.68
Present	51 (4.30%)	40 (19.61%)	11 (5.39%)	19 (27.50%)	13 (18.84%)	6 (8.70%)	
WBC, 10^9^/L	7.48 (5.95–9.03)	7.56 (6.10–8.97)	6.93 (5.67–9.80)	7.21 (5.73–9.50)	7.09 (5.56–9.11)	8.29 (6.99–10.45)	0.80
Neutrophils, 10^9^/L	4.86 (3.79–6.51)	4.99 (3.79–6.35)	4.80 (3.67–7.50)	4.80 (3.60–6.70)	4.30 (3.32–5.70)	6.60 (4.55–7.90)	0.90
Lymphocytes, 10^9^/L	1.54 (1.14–1.96)	1.63 (1.20–2.11)	1.30 (1.10–1.65)	1.80 (1.10–2.10)	1.85 (1.24–2.22)	1.07 (0.85–1.50)	0.80
Monocytes, 10^9^/L	0.54 (0.42–0.70)	0.54 (0.43–0.70)	0.54 (0.40–0.7)	0.50 (0.40–0.70)	0.50 (0.40–0.70)	0.60 (0.50–0.70)	0.79
NLR	3.15 (2.10–4.57)	2.98 (2.01–4.38)	3.45 (2.30–6.67)	2.75 (1.96–5.00)	2.39 (1.69–3.94)	4.38 (3.16–9.21)	0.66
LMR	2.82 (1.92–4.00)	2.89 (2.00–4.04)	2.33 (1.85–3.41)	3.00 (1.94–4.48)	3.60 (2.05–4.80)	1.80 (1.20–2.84)	0.37
PLR	172.04 (126.97–250.69)	172.14 (127.27–254.09)	171.93 (133.07–249.52)	151.11 (113.81–244.44)	148.39 (110.16–228.89)	157.50 (125.90–261.11)	0.21
PLT, 10^9^/L	272.50 (219.25–356.00)	288.00 (223.00–371.00)	232.00 (197.50–280.00)	258.00 (207.00–327.00)	270.5 (211.5–325.75)	230.00 (161.00–289.50)	0.04
PT, s	11.70 (11.13–12.10)	11.60 (11.10–12.10)	11.70 (11.30–12.10)	11.60 (10.85–12.10)	11.50 (10.80–12.10)	11.80 (11.45–12.05)	0.50
APTT, s	26.15 (24.20–27.98)	26.10 (24.25–27.50)	26.8 (24.0–29.50)	25.50 (23.45–27.45)	24.80 (23.30–27.05)	27.50 (25.7–30.85)	0.28
Fbg, g/L	4.55 (3.59–5.75)	4.68 (3.58–5.81)	4.43 (3.64–5.22)	3.80 (3.29–5.02)	3.68 (3.24–5.17)	4.45 (3.44–4.98)	0.59
TT, s	17.10 (16.30–17.88)	17.00 (16.20–17.75)	17.60 (16.70–18.05)	17.40 (16.80–18.00)	17.45 (16.80–18.10)	17.10 (16.60–17.65)	0.20
CD3 + cells, %	68.67 (60.70–75.67)	69.28 (61.50–76.39)	66.25 (59.27–73.64)	70.50 (63.40–76.10)	69.40 (62.68–73.94)	76.40 (69.80–78.33)	0.41
CD3 + CD4 + cells, %	38.59 (30.20–43.30)	40.07 (31.57–44.90)	34.71 (28.05–40.08)	35.95 (29.25–44.45)	35.63 (29.18–41.93)	44.90 (32.00–46.70)	0.71
CD3 + CD8 + cells, %	25.57 (22.08–33.18)	25.86 (20.69–33.18)	25.35 (22.38–33.38)	26.90 (22.60–34.20)	26.90 (22.45–35.08)	28.80 (22.13–34.93)	0.28
B cells, %	8.02 (5.93–11.43)	9.11 (6.25–11.74)	7.15 (4.80–9.93)	9.15 (6.34–12.60)	9.15 (6.50–14.45)	8.75 (5.30–11.13)	0.36
CD3-CD16 + CD56 + cells, %	18.05 (12.93–25.80)	17.03 (11.67–24.28)	21.20 (16.58–28.40)	17.65 (11.65–20.99)	18.50 (13.45–21.32)	12.10 (9.18–17.48)	0.17
Treg cells, %	23.45 (17.55–31.25)	22.95 (17.13–28.45)	25.00 (19.18–37.43)	20.15 (14.90–25.70)	18.60 (14.13–24.50)	31.75 (20.75–40.28)	0.13
CD8 + CD25 + cells, %	7.65 (4.28–11.18)	8.95 (6.1–12.43)	5.25 (3.15–8.83)	7.15 (4.75–10.30)	725 (4.93–10.48)	5.90 (4.55–8.45)	0.91
TNM stage, n (%)							
Stage II	5 (2.45%)	2 (0.98%)	3 (1.47%)	0	0	0	
Stage III	55 (26.96%)	50 (24.51%)	5 (2.45%)	21 (30.43%)	18 (26.09%)	3 (4.35%)	0.51
Stage IV	144 (70.59%)	105 (51.47%)	79 (38.73%)	48 (69.57%)	36 (52.17%)	12 (17.39%)	0.78

### Independent prognostic factors selection

**Fig. 1:**
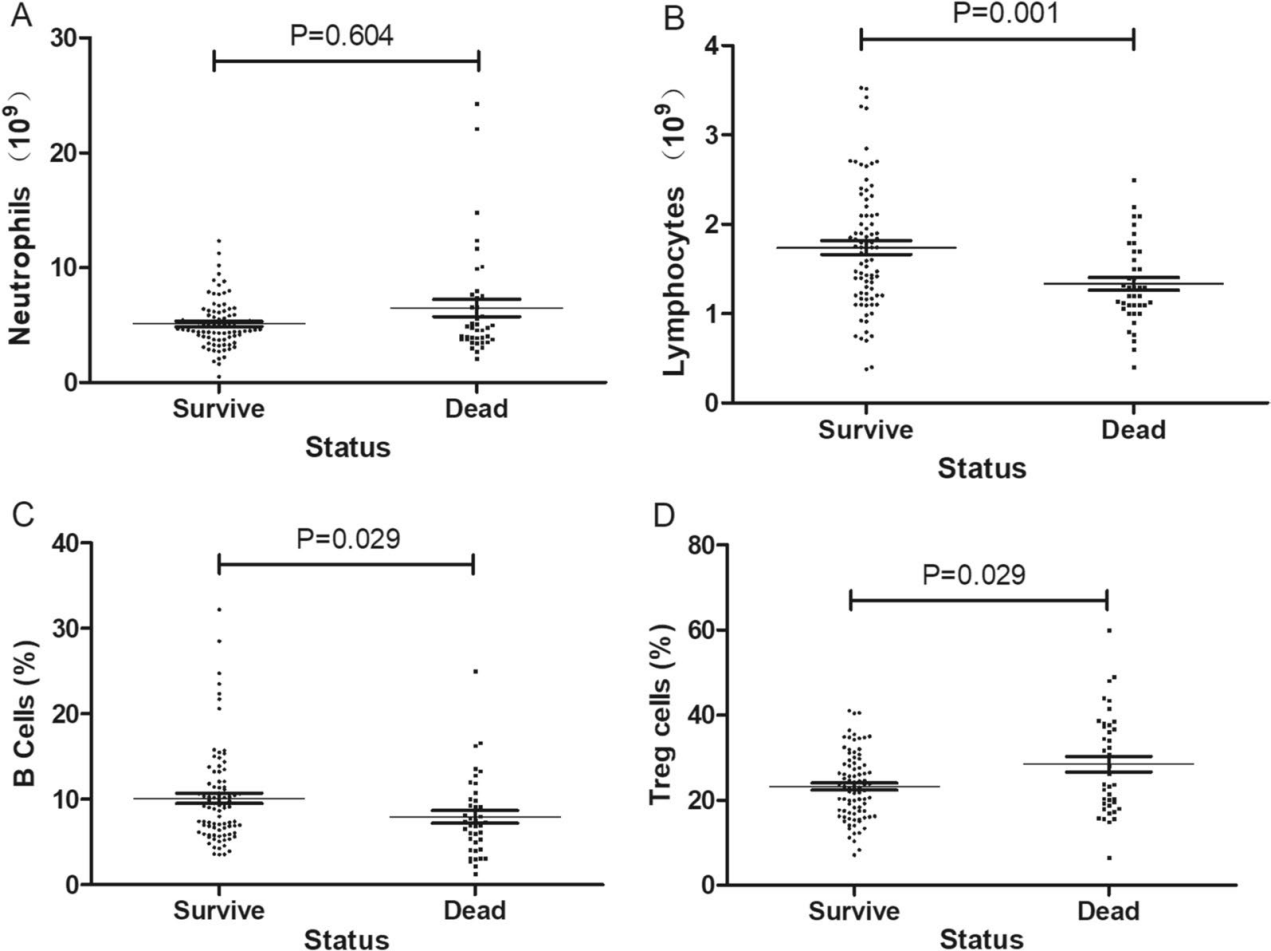
The number of neutrophils **A**, lymphocytes **B** and the proportion of B cells **C**, Treg cells **D** of survival and dead patients in training cohort

**Fig. 2:**
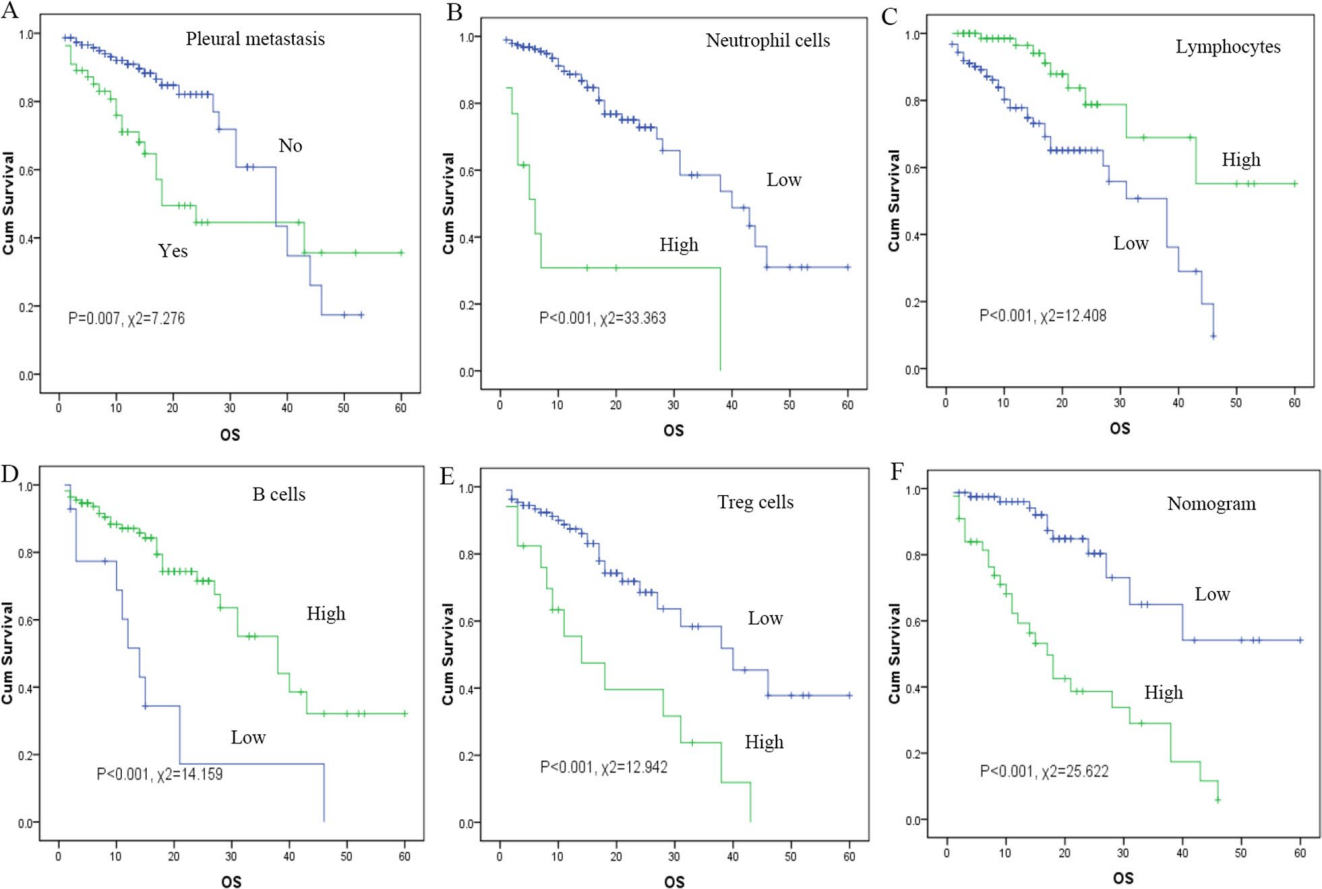
Kaplan–Meier curve analysis. Survival curves of pleural metastasis **A**, neutrophils **B**, lymphocytes **C**, B cells **D**, Treg cells **E** and nomogram **F** in the training cohort

**Fig. 3:**
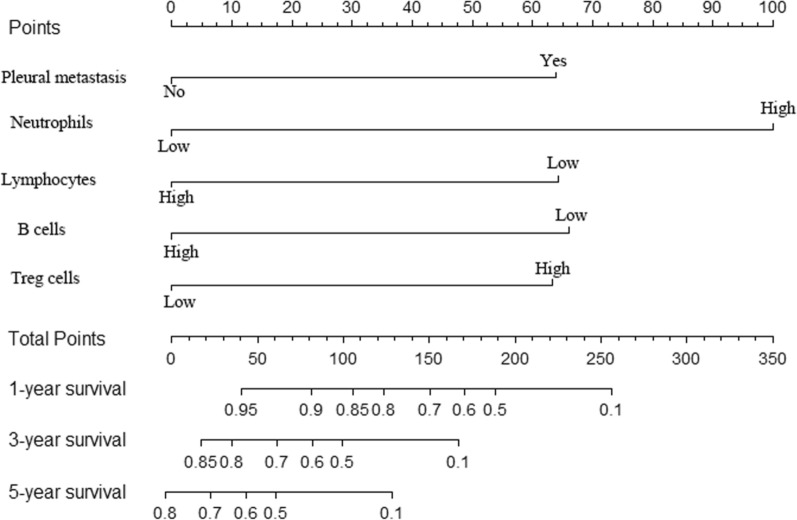
The developed nomogram for predicting overall survival. The nomogram was developed based on the training cohort, with the use of pleural metastasis, neutrophils, lymphocytes, B cells and Tregs

**Fig. 4:**
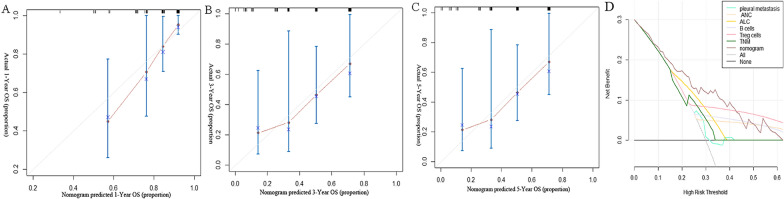
Calibration curves and DCA curves. Calibration curves for 1-year **A**, 3-year **B**, and 5-year **C** OS in the training cohort. The predicted possibility of the year-specific OS rate is indicated in the x-axis, and the actual possibility of the year-specific OS rate is indicated in the y-axis. DCA curves in the training cohort **D**. Clinical usefulness of different predictive systems in predicting overall survival at various time points. The y-axis represents net benefit. The x-axis shows threshold probability. The brown line displays the benefit of the developed nomogram. The light blue line displays the benefit of pleural metastasis. The faint yellow line displays the benefit of neutrophils. The deep yellow line displays the benefit of lymphocytes. The grey line displays the benefit of the B cells. The pink line displays the benefit of Treg cells. The green line displays the benefit of TNM

**Fig. 5:**
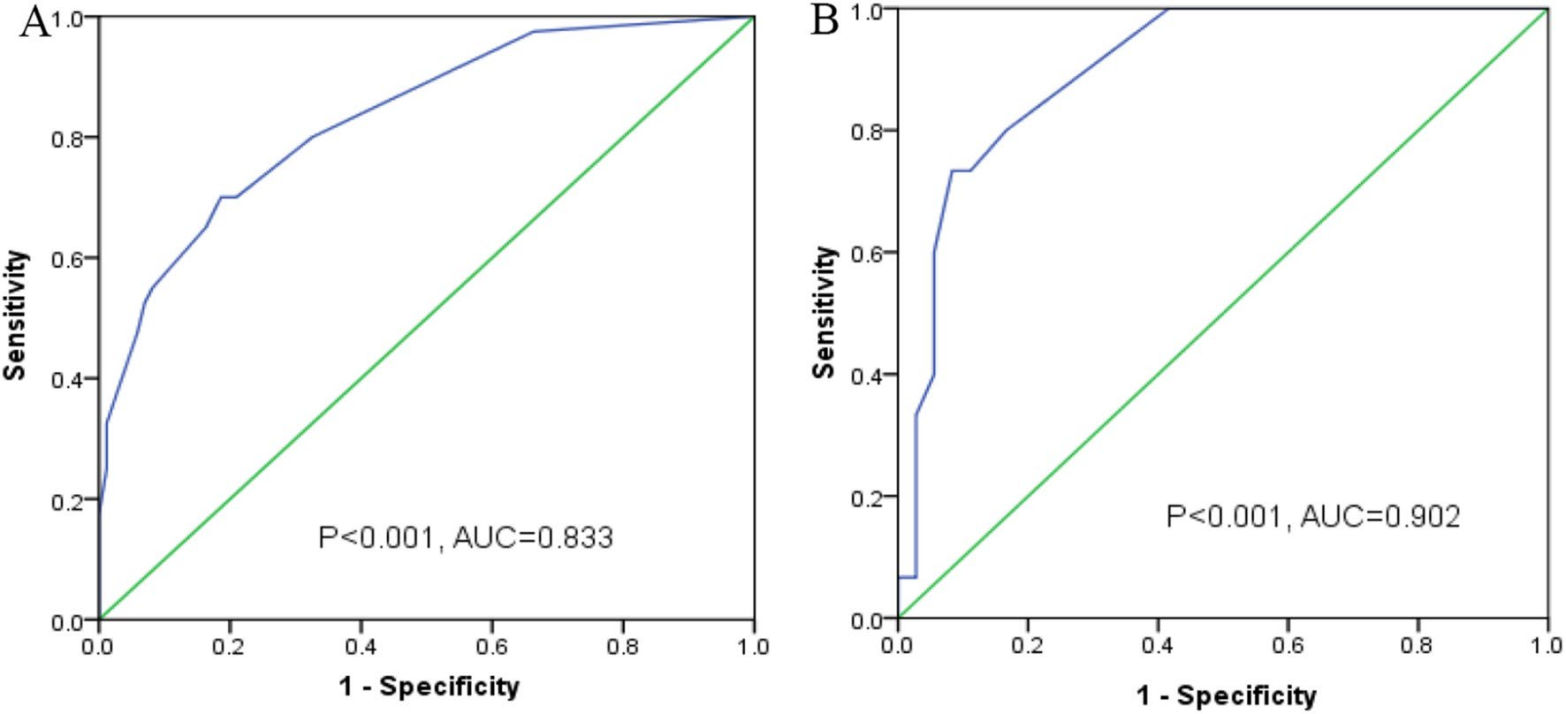
ROC curves. ROC curves for training cohort (**A**) and validate cohort (**B**)

10 factors of all the demographic, laboratory examination and clinicopathological variables were selected out in univariate analysis. Pleural metastasis, WBC, neutrophils, lymphocytes, NLR, LMR, CD3 + CD4 + cells, CD3−CD16 + CD56 + cells, B cells and Treg cells were significantly correlated with the prognostic of NSCLC patients. All significant prognostic factors were entered into the further multivariate cox hazards analysis, which revealed pleural metastasis, ANC, ALC, B cells and Treg cells were independent prognostic factors (Table 2). Pleural metastasis, ANC > 10.00, ALC < 1.71, B cells < 4.23, Treg cells > 35.8 being adverse factors correlating with worse prognostic and shorter survival time (Figs. 1, 2, 3, 4 and 5).

**Table 2:** Univariate and multivariate cox hazards analysis of the primary cohort

Characteristic	Univariate analysis		Multivariate analysis	
	HR (95% CI)	P	HR (95% CI)	P
Age*, n (%)*				
< 59 vs. ≥ 59	0.854 (0.478–1.528)	0.596		
Sex*, n (%)*				
Male vs. Female	1.043 (0.515–2.113)	0.906		
BMI*, n (%)*				
< 18.5 vs. 18.5–24 vs. ≥ 24	1.085 (0.646–1.821)	0.758		
Alcohol*, n (%)*				
No/Yes	0.946 (0.441–2.031)	0.887		
Smoking*, n (%)*				
No/Yes	1.360 (0.758–2.442)	0.303		
Histology*, n (%)*				
Adenocarcinoma / Squamous cell carcinoma	1.646 (0.914–2.963)	0.097		
No. of metastatic sites*, n (%)*				
< 2 vs. ≥ 2	1.038 (0.563–1.913)	0.904		
Pleural metastasis*, n (%)*				
Absent vs. Present	2.172 (1.214–3.883)	0.009	2.980 (1.521–5.837)	0.001
Lung metastasis*, n (%)*				
Absent vs. Present	0.688 (0.365–1.298)	0.248		
Brain metastasis*, n (%)*				
Absent vs. Present	0.784 (0.309–1.989)	0.609		
Liver metastasis*, n (%)*				
Absent vs. Present	1.296 (0.603–2.782)	0.506		
Bone metastasis*, n (%)*				
Absent vs. Present	1.002 (0.509–1.973)	0.995		
WBC*, 10*^*9*^*/L*				
< 11.80 vs. ≥ 11.80	4.185 (1.989–8.809)	< 0.001		
Neutrophils*, 10*^*9*^*/L*				
< 10.00 vs. ≥ 10.00	6.590 (3.130–13.877)	< 0.001	5.139 (2.081–12.691)	< 0.001
Lymphocytes*, 10*^*9*^*/L*				
< 1.71 vs. ≥ 1.71	0.293 (0.141–0.609)	0.001	0.331 (0.142–0.771)	0.010
Monocytes*, 10*^*9*^*/L*				
< 0.43 vs. ≥ 0.43	0.941 (0.513–1.728)	0.846		
NLR				
< 4.90 vs. ≥ 4.90	3.439 (1.854–6.381)	< 0.001		
LMR				
< 2.76 vs. ≥ 2.76	0.367 (0.200–0.673)	< 0.001		
PLR				
< 81.98 vs. ≥ 81.98	0.697 (0.215–2.263)	0.548		
PLT*, 10*^*9*^*/L*				
< 273.00 vs. ≥ 273.00	0.612 (0.317–1.185)	0.145		
PT*, s*				
< 11.30 vs. ≥ 11.30	1.726 (0.819–3.637)	0.152		
APTT*, s*				
< 27.50 vs. ≥ 27.50	1.738 (0.923–3.273)	0.087		
Fbg*, g/L*				
< 4.82 vs. ≥ 4.82	0.892 (0.450–1.769)	0.743		
TT*, s*				
< 17.60 vs. ≥ 17.60	1.121 (0.583–2.158)	0.732		
CD3 + cells*, %*				
< 67.20 vs. ≥ 67.20	0.694 (0.371–1.298)	0.252		
CD3 + CD4 + cells*, %*				
< 40.40 vs. ≥ 40.40	0.451 (0.207–0.984)	0.045		
CD3 + CD8 + cells*, %*				
< 16.70 vs. ≥ 16.70	2.809 (0.675–11.697)	0.156		
B cells*, %*				
< 4.23 vs. ≥ 4.23	0.275 (0.133–0.568)	< 0.001	0.329 (0.151–0.714)	0.005
CD3-CD16 + CD56 + cells*, %*				
< 16.10 vs. ≥ 16.10	2.229 (1.056–4.705)	0.035		
Treg cells*, %*				
< 35.80 vs. ≥ 35.80	3.197 (1.628–6.280)	0.001	2.934 (1.478–5.826)	0.002
CD8 + CD25 + cells*, %*				
< 5.95 vs. ≥ 5.95	0.539 (0.284–1.024)	0.059		
TNM stage				
Stage II vs stage III vs stage IV	1.574 (0.782–3.167)	0.204

### Prognostic nomogram

All independent prognostic factors based on the multivariate Cox regression analysis were incorporated to build a predictive model, which was visualized in the form of a nomogram. Each predictor in the nomogram was assigned a score, ranging from 0 (lowest risk) to 100 (highest risk). The nomogram showed that neutrophils  > 11.8 was the most meaningful risk factors, while pleural metastasis, lymphocytes  < 1.71, B cells  < 4.23 and Treg cells  > 35.8 showed a moderate effect on patient’s survival. By summing up total scores and drawing a straight line through the location of total points, we could obtain the estimated probability of 1-year 3-year and 5-years survival of NSCLC patients.

### Clinical utility of the nomogram

The C-index analysis indicated that the nomogram provided training cohort and validating cohort C-indexes of 0.808 and 0.741, respectively. The calibration curve of 1-year, 3-year and 5-year has good consistency with the 45-degree ideal line. The nomogram shows good accuracy in predicting the prognosis of NSCLC patients. To verify the clinical usefulness of the model and to prove the necessity of nomogram, decision-curve analysis(DCA) was conduct. In DCA curves, the benefit of the model was compared with pleural metastasis, neutrophils, lymphocytes, B cells, Treg cells and TNM stage. During all the curves, the new model showed the largest net benefits in predict survival.

### Performance of the nomogram

And receiver operation characteristic curve of both training cohort and validation cohort demonstrated the prediction value of the model with the area under curve. The area under the curve (AUC) are 0.833 (P < 0.001) in training cohort and 0.908 (P < 0.001) in validation cohort. The ROC curve demonstrated the prediction value of the model.

## Discussion

In the present study, we described the clinical characteristics, risk factors and constructed a model of the outcome of NSCLC patients treated with anti-PD-1/PD-L1 plus bevacizumab to provide a theoretical basis for better prognosis. In our study, several factors showed significant correlation with OS, which include neutrophils, lymphocytes, pleural metastasis, B cell and Treg cell. These predictive factors used in nomogram can be easily acquired from clinical information and laboratory information system, making it feasible for application in clinical practice.

As adding bevacizumab to immunotherapy has become a common combined immunotherapy strategies in NSCLC treatment, an accurate and simple tool to predict prognosis is an urgent need. In this study, we established and validated a nomogram for NSCLC patients treated with anti-PD-1/PD-L1 plus bevacizumab to rapidly predict the long-term prognosis by combining simple clinicopathological factors and hematological indicators. Due to the poor long-term survival of NSCLC patients, an accurate and economical prediction of prognosis in patients after diagnosis is of increasing clinical significance. We anticipate that this practical predictive tool can potentially guide individualized therapy, as doctors can predict the prognosis of patients and the early intervention can be given presciently.

TME is a complex system which contains tumor cells, immune cells [T-cells, B-cells, dendritic cells, myeloid-derived suppressor cells (MDSCs), tumor-associated macrophages (TAMs), tumor-associated neutrophils (TANs)], carcinoma-associated fibroblasts (CAFs), vascular system and extracellular matrix components [8]. In contrast to traditional chemotherapy, the immunotherapy mainly take effect through the immune cells within or outside the TME to specially recognize and attack the tumor cells, which theoretically makes the immunotherapy higher specificity and lower side effect [9]. Anti-PD-1/PD-L1 could interrupt multiple signal pathways which relate to the function of T cells and enhance anti-tumor immunity [10]. Anti-VEGF, antibody act on an angiogenesis stimulator, enhance tumor immunity by accelerating the maturation of dendritic cells, and inhibit immunosuppressive cells such as regulatory T cells [11]. Current combination therapies with ICI and anti-VEGF showed favorable changes in the TME.

Traditionally, neutrophils, despite widespread in the TME, are seemed to be an indicator of innate immune response [12]. In our study, high neutrophils represent high risk in the prognosis of NSCLC patients. Contrary to previous view, neutrophils may play an important role in tumor progression. Lin et al. demonstrated that tumor-infiltrating neutrophils promote tumor growth in Pancreatic ductal adenocarcinoma. Especially, they took high intratumor neutrophils and high IL-8 levels for poor outcomes of immune checkpoint inhibitors therapy and worse survival in patients with advanced cancers [13]. In other study, Farnaz et al. explained the role of neutrophils as a pro-metastatic agent in breast cancer and considered the increasing neutrophils in tumors as a failed immune response to cancer [14]. In another large 16-year cohort, high neutrophil-to-lymphocyte ratio was closed to increased risk of lung cancer mortality in low-risk individuals [15].

Lymphocyte is always closed related to immune system. High lymphocyte is to be a positive prognostic factors of a NSCLC patients in our research. Likewise, Kobayashi et al. reported that low lymphocyte was a more valuable predictor of poor prognosis in node-negative NSCLC [16]. And the results of Huang' study suggested that a high absolute peripheral lymphocyte count is an independent protective factor, and it had a high clinical benefit for patients with lung cancer [17].

As a member of CD4 + T cells, Tregs express special markers including CD25 and regulate suppressive signals. Indeed, Tregs are on behalf of a risk sign to cancer patient. Several studies revealed the regulatory function of Tregs in tumor behavior in the TME [18, 19]. While suppressing the over-reactive immune response in autoimmune disease, Tregs in TME prevent the effective response of cytotoxic T lymphocytes (CTLs) on tumor cells. Xu et al. considered the function of Tregs is closely associated with the prognosis of patients [9].

CD19 is the most specific and common marker of B cells. B cell is a protective factor in NSCLC patients in our study. It is generally accepted that B cell produces antibody and cytokines to regulate immune responses and inflammation as well as inducing T cell activation and proliferation via antigen presentation [20]. A study which conducted a single-cell RNA-seq analysis demonstrated that the naïve-like B cells suppress the growth of lung cancer cells in NSCLC patients [21]. Another recent study has revealed B cells are related with favorable prognosis of NSCLC [22].

Pleural invasion is always seemed to be an independent risk factor and an important prognostic factor of NSCLC [23]. In the 8th edition of the TNM classification for NSCLC, if a tumor shows ipsilateral pleural dissemination, it increases the T descriptor from T1 to T2 and upstages a tumor from stage IA to stage IB, no matter how small the size is [24]. Studies from different groups showed that NSCLCs with pleural transfusion is more likely to be poor differentiated, more invasive and have larger tumor size [25–27]. These results accord closely with our analysis.

This study had several limitations. Firstly, the volume of NSCLC patients is large in our hospital but the number of patients using bevacizumab is limited so that the subjects included is limited. Secondly, it was a retrospective study. This may have influenced the power of the analysis to build model. Further prospective studies are needed.

In conclusion, to our knowledge, this is the first study in China to set a model to predict the prognosis of NSCLC patients treated with anti-PD-1/PD-L1 plus bevacizumab, which provides a useful basis for the treatment of NSCLC patients. Compared with the commonly used TNM stage, we established a particular model for certain people. Compared to conventional testing techniques, such as immunohistochemistry, which is expensive and time-consuming, we selected clinicopathological factors and hematological indicators. Physicians could rapidly predict the prognosis of patients and propose prognosis-based therapeutic protocol for NLCSC patients. Additional studies would be required to explore whether the nomogram can also be applied to predict the effective of treatment.

## Conclusion

In summary, our study pay attention to NSCLC patients treated with PD-1/PD-L1 antibody plus antiangiogenic drugs and build a new nomogram to predict long-time OS of these patients. The nomogram may help clinicians to accurately estimate long-time OS of NSCLC patients treated with PD-1/PD-L1 antibody and antiangiogenic drugs in the early stage of treatment.

## Data Availability

The original contributions presented in the study are included in the article. Further inquiries can be directed to the corresponding author.

## Abbreviations

PD-1/PD-L1: Programmed cell death protein 1/programmed death-ligand 1
NSCLC: Non-small cell lung cancer
ICIs: Immune checkpoint inhibitors
VEGF: Vascular endothelial growth factor
OS: Overall survival
EMR: Electronic medical record system
LIS: Laboratory information system
ANC: Absolute neutrophil count
ALC: Absolute lymphocyte count
HR: Hazard ratio
NLR: Isneutrophil–lymphocyte ratio
LMR: Lymphocyte-monocyte ratio
PLR: Platelet to lymphocyte ratio
ROC: Receiver operation characteristic curve
AUC: Area under curve
TME: Tumor microenvironment
MDSCs: Myeloid-derived suppressor cells
TAMs: Tumor-associated macrophages
TANs: Tumor-associated neutrophils
CAFs: Carcinoma-associated fibroblasts
CTLs: Cytotoxic T lymphocytes
